# Impact of *RRP1B* Variants on the Phenotype, Progression, and Metastasis of Cervical Cancer

**DOI:** 10.3390/cancers16071250

**Published:** 2024-03-22

**Authors:** Eglė Balčiūnienė, Arturas Inčiūra, Elona Juozaitytė, Rasa Ugenskienė

**Affiliations:** 1 Institute of Oncology, Lithuanian University of Health Sciences, LT-50161 Kaunas, Lithuania; arturas.inciura@lsmu.lt (A.I.); elona.juozaityte@lsmu.lt (E.J.); rasa.ugenskiene@lsmu.lt (R.U.); 2 Department of Genetics and Molecular Medicine, Lithuanian University of Health Sciences, LT-50161 Kaunas, Lithuania

**Keywords:** cervical cancer, *RRP1B*, polymorphisms, genotype, haplotype, metastasis, survival

## Abstract

**Simple Summary:**

Metastasis, a critical aspect of oncologic diseases, is intricately governed by genetic factors. This article delves into the role of the ribosomal RNA processing 1 homolog B (*RRP1B*) gene in metastasis regulation, investigating its implications in human cervical cancer. We analyzed five *RRP1B* polymorphisms in 172 cervical cancer patients to understand their associations with disease characteristics and survival. Certain variations were linked to decreased tumor size, reduced metastasis risk, and improved overall survival, suggesting their potential as markers for predicting prognosis in cervical cancer.

**Abstract:**

Metastasis is a key determinant of cancer progression, influenced significantly by genetic mechanisms. RRP1B, primarily a nucleolar protein, emerges as a suppressor of metastasis, forming alliances with various cellular components and modulating gene expression. This study investigates the involvement of the ribosomal RNA processing 1 homolog B (*RRP1B*) gene in metastasis regulation in cervical cancer. Through a comprehensive analysis of 172 cervical cancer patients, we evaluated five *RRP1B* single nucleotide polymorphisms (SNPs) (rs2838342, rs7276633, rs2051407, rs9306160, and rs762400) for their associations with clinicopathological features and survival outcomes. Significant associations were observed between specific genetic variants and clinicopathological parameters. Notably, the A allele of rs2838342 was associated with reduced odds of advanced tumor size, worse prognosis, and, preliminarily, distant metastasis, while the T allele of rs7276633 correlated with a decreased risk of higher tumor size and worse prognosis. Additionally, the C allele of rs2051407 demonstrated protective effects against larger tumors, metastasis, and adverse prognosis. The rs9306160 C allele exhibited a protective effect against metastasis. The rs762400 G allele was significant for reduced tumor size and metastasis risk. Furthermore, the rs2838342 A allele, rs7276633 T allele, rs2051407 C allele, and rs762400 G allele were associated with improved overall survival, demonstrating their potential significance in predicting prognoses in cervical cancer. Linkage disequilibrium and haplotypes analysis enabled us to evaluate the collective effect of the analyzed SNPs, which was in line with the results of allelic models. Our findings underscore the clinical relevance of *RRP1B* SNPs as prognostic markers in cervical cancer, shedding light on the intricate interplay between genetic factors and disease-progression dynamics. This research provides critical insights for future investigations and underscores the importance of incorporating *RRP1B* SNP detection into prognostic-assessment tools for accurate prediction of disease outcomes in cervical cancer.

## 1. Introduction

Metastasis stands as a paramount and intricate phenomenon in the domain of oncologic diseases. Earlier investigations have ascertained the noteworthy impact of the genetic context in which tumors originate on their proclivity for metastasis. Predictive human gene-expression profiles associated with metastasis exhibit their presence not solely in mouse tumors featuring varying metastatic capacities, but they also display a discernible correlation with the inherent genetic backdrop. It is suggested that the genesis of human metastasis-predictive gene expression signatures may be markedly propelled by the genetic background, eclipsing the influence of acquired somatic mutations [1,2,3,4,5,6]. The capacity to discern individuals at an elevated risk of disseminated disease precisely during the clinical manifestation of primary cancer holds the potential for a substantial paradigm shift in cancer management.

Employing a meticulously characterized transgenic model of mouse mammary tumorigenesis, the ribosomal RNA-processing 1 homolog B (*RRP1B/KIAA0179)* gene has been pinpointed as a potential modifier QTL gene impacting metastasis efficiency [7,8,9]. RRP1B is primarily identified as a nucleolar protein and is also a nuclear membrane-associated protein, although it has been reported in multiple cellular locations. *The RRP1B* gene is located on Chromosome 21q22.3, and the protein contains 758 amino acids. Previous investigations have revealed that *RRP1B* forms a binding alliance with the metastasis-modulating factor GTPase activator *SIPA1* [10,11]. Simultaneously, in vitro, using mouse and human metastasis gene-expression data, *RRP1B* expression was found to be associated with extracellular matrix gene (ECM) expression and to be a germline regulator of ECM genes, which are recognized as metastasis-predictive components with different regulation in metastasis-prone tumors. The ectopic expression of RRP1B inhibited tumor growth and metastasis in the highly metastatic mouse mammary tumor cell line. The significance of *RRP1B* was underscored by the discovery that germline polymorphisms (SNPs) within the human *RRP1B* consistently correlate with clinical breast cancer outcomes and survival [8,12].

*RRP1B* upregulation is associated with metastasis suppression. *RRP1B* physically interacts with many nucleosome-binding factors. The primary outcome of transcriptional repression is RRP1B binding to chromatin, and it occupies loci with decreased gene expression. *RRP1B* orchestrates the regulation of metastasis-associated gene expression through its interaction with the transcriptional corepressors tripartite motif-containing protein 28 (TRIM28) and heterochromatin protein 1-α (HP1α) by recruiting chromatin-modifying enzymes. RRP1B influences histone methylation changes [12,13]. RRP1B suppresses metastatic progression while also modulating the expression of alternative mRNA isoforms through interactions with the splicing regulator and oncoprotein SRSF1 [14]. Further experimentation demonstrated that RRP1B interacts with protein phosphatase 1 (PP1), whose functions are implicated in tumorigenesis, the tumor microenvironment, and the metastatic cascade, and it regulates nucleolar phosphorylation signaling [15,16,17]. *RRP1B* enhances DNA damage-induced apoptosis by functioning as a transcriptional coactivator for proapoptotic target genes under the regulation of the transcriptional activator E2F1 [18].

RRP1B associates with the nucleolar phosphoprotein NPM1, participating in cellular proliferation, growth-suppression pathways, and the apoptotic response to oncogenic stimuli such as DNA damage and hypoxia. NPM1 is implicated in tumorigenesis [12,19,20]. Furthermore, RRP1B interacts with the protein BRD4, a transcriptional and epigenetic regulator that holds a pivotal role in cancer development [21,22,23]. *RRP1B* can upregulate the expression of claudin-1 by depleting DOCK1 and increase cell viability and motility of claudin-low breast cancer cells [24]. It is proposed that *RRP1B* is targeted by miR-320a and contributes to cancer survival [25].

Various studies underscore the multifaceted nature of how *RRP1B* governs both transcription and metastasis. The dysregulation of *RRP1B* exerts a net effect on multiple pathways and biological processes, underscoring the complexity of its influence on metastasis and prognostic gene expression.

While the molecular understanding of *RRP1B* as a potential modifier of metastasis is present, there is a scarcity of reports concerning the impact of host genetic factors on various cancer progressions and metastases.

Cervical cancer (CC) ranks among the most frequently diagnosed cancers and stands as the foremost cause of cancer-related mortality in women on a global scale. As reported by the World Health Organization (WHO), it holds the position as the fourth-most prevalent cancer affecting women worldwide. In 2020, the World Health Organization reported an estimated 604,000 new cases and 342,000 deaths worldwide [26]. While cervical cancer remains a leading cause of cancer-related mortality among women in sub-Saharan Africa, with incidence rates as high as 40 per 100,000 women [27], the burden of the disease in Europe is notable for its variance between countries. The Human papillomavirus (HPV) infection is the primary risk factor for cervical cancer, with certain high-risk HPV types, notably HPV 16 and 18, being responsible for the majority of cases [28,29]. Advances in screening methods, such as HPV testing and Pap smears, have significantly improved early detection and prevention efforts, leading to a decrease in cervical cancer incidence and mortality rates in many countries [30]. Vaccination against HPV has emerged as a powerful strategy for cervical cancer prevention, with vaccines targeting the most oncogenic HPV types, demonstrating high efficacy in preventing HPV infection and subsequent cervical lesions [31]. While many countries in Western Europe have implemented organized screening initiatives and achieved substantial reductions in cervical cancer burden, disparities persist in Eastern and Southern Europe, where access to screening services may be limited and screening uptake rates remain suboptimal. Recent data from the European Centre for Disease Prevention and Control (ECDC) indicate that although cervical cancer incidence rates have been declining in most European countries, mortality rates remain a concern, particularly in regions with lower screening coverage and vaccination rates [32]. Despite these challenges, recent advancements in molecular biology and genetics offer promising avenues for improving cervical cancer prevention and treatment. Understanding the genetic determinants of cervical cancer susceptibility, progression, and treatment response is crucial for developing targeted interventions and personalized treatment strategies. Emerging research is focused on further understanding the molecular mechanisms underlying cervical cancer development and progression, as well as identifying novel biomarkers and therapeutic targets to improve patient outcomes.

Previous studies have indicated that inherited polymorphisms are associated with specific tumor characteristics and subsequent outcomes in human cancer. Recognizing the potential impact of germline polymorphisms on disease pathomorphological features and disease progression, we examined five single nucleotide polymorphisms (SNPs) (rs2838342, rs7276633, rs2051407, rs9306160, and rs762400) within the *RRP1B* gene among cervical cancer patients. Our investigation aimed to elucidate their effect on the clinical manifestations and outcomes of the disease.

## 2. Materials and Methods

### 2.1. Study Subjects

The retrospective cohort study of adult patients with cervical cancer was approved by the Kaunas Regional Biomedical Research Ethics Committee (No. BE-2-10 and P1-BE-2-10/2014). All the patients were investigated at the Hospital of Lithuanian University of Health Sciences Kaunas Clinics in Kaunas, Lithuania from October 2014 to August 2020. A total of 172 patients with Stages I–IV cervical cancer were consecutively enrolled, with their diagnoses confirmed through clinical (gynecological and radiological examinations) and histological (cervical biopsies) assessments. Inclusion criteria were the availability of complete data on clinicopathological characteristics and the patient’s written consent to participate. Exclusion criteria encompassed the presence of other malignancies, significant comorbidities, and incomplete medical records, which were used to extract clinical and pathological features, as well as details about the disease course. The blood samples were collected from peripheral veins for further genetic testing. Cancer treatment was administered following institutional guidelines and in accordance with international standards. The follow-up period was extended until November 2020.

### 2.2. SNP Selection

Genotype information was derived from established online repositories, including The International HapMap Project (http://www.HapMap.org, accessed on 1 September 2023) and the 1000 Genomes Project (http://www.1000genomes.org, accessed on 1 September 2023). The criteria employed for the selection of *RRP1B* Single Nucleotide Polymorphisms (SNPs) were comprehensive. These criteria involved the prerequisite that these SNPs had been previously identified in diverse populations, showcasing associations with the outcomes of various diseases as reported in scientific literature. Moreover, our analysis specifically targeted SNPs that had not been extensively investigated within the context of cervical cancer patients, thereby exploring new avenues of genetic inquiry. Additionally, SNPs under consideration were required to exhibit a minor allele frequency (MAF) equal to or greater than 5% within the European population. This criterion was pivotal in ensuring that the selected SNPs had a sufficiently substantial presence to be statistically significant. Finally, we also considered the potential functional relevance of these SNPs, exploring whether they might be involved in regulating key biological processes. As a point of reference, Table 1 provides a comprehensive listing of the candidate SNPs (rs2838342, rs7276633, rs2051407, rs9306160, and rs762400), their locations, and MAF within the European population data from the 1000 Genomes Project Phase 3 database.

### 2.3. Methods

All carcinoma cases were staged according to the guidelines set forth by the International Federation of Gynecology and Obstetrics (FIGO). Tumor grading was determined based on architectural and cytologic (nuclear) criteria. This analysis incorporated clinicopathological features, including age at the time of diagnosis, tumor size (T), lymph node involvement (N), metastasis spread (M), stage, degree of differentiation (G), response to treatment, presence of disease progression, and patient mortality.

The DNA-extraction process involved the isolation of genetic material from leukocytes in peripheral venous blood samples, which were initially collected in ethylenediaminetetraacetate (EDTA) vacuum tubes and subsequently stored in a laboratory biobank at −20 °C. Genomic DNA extraction was conducted utilizing a genomic DNA purification kit provided by Thermo Fisher Scientific Baltics, based in Vilnius, Lithuania. Genotyping of five selected SNPs within the *RRP1B* gene was conducted at the Institute of Oncology, Lithuanian University of Health Sciences. This was achieved using TaqMan^®^ probe SNP genotyping assays, also sourced from Thermo Fisher Scientific in Lithuania. Molecular genetic analyses were performed employing the real-time polymerase chain reaction (RT-PCR) method, which is designed to amplify specific DNA segments as per the established protocol.

In our comprehensive investigation, we delved into the potential interconnections between SNPs in the genotype and allelic models, and the intricate landscape of tumor clinicopathological features. These attributes encompass the patient’s age (categorized into age ≤50 and age >50), the tumor’s size (distinguished as T1–T2 and T3–T4), the status of pathological regional lymph nodes (delineated as N0 and N1), the presence of distant metastasis (defined by M0 or M1), the tumor’s grade (G1 + G2 or G3), the disease stage (categorized as Stages I–II and Stages III–IV), and the overall disease prognosis (specifically, the worse prognosis: T3–T4 + G3 versus T1–T2 + G1–G2). Furthermore, the study extended its scrutiny to encompass clinical outcomes, specifically progression-free survival (PFS) and overall survival (OS). Within the patient cohort, PFS was computed commencing from the date of diagnosis until the point of local disease spread or the occurrence of distant metastasis/metastasis spread. In parallel, OS was calculated from the date of diagnosis to the date of the patient’s demise. Haploview v4.1 software was utilized to assess linkage disequilibrium (LD) among SNPs and generate LD plots (available at http://www.broad.mit.edu/mpg/haploview/, accessed on 5 January 2024). Haplotypes were inferred from the analyzed SNPs using Bayesian methods through the phase software v2.1 (Department of Statistics, University of Washington, Seattle, WA, USA). Finally, we analyzed the associations of haplotypes with clinical manifestations of the disease and survival outcomes. These findings are instrumental in our quest to elucidate the potential genetic factors that may exert influence over these pivotal facets of the disease’s clinical intricacies.

### 2.4. Statistical Analysis

The identified SNPs were subsequently integrated into a comprehensive statistical analysis, encompassing both genotype and allelic models. The statistical evaluation was conducted using SPSS version 25.0 (SPSS Inc., Chicago, IL, USA). To investigate the associations between genotypes, alleles, and tumor characteristics, statistical tests, including Pearson’s Chi-square and Fisher’s Exact tests, were employed. In order to present a robust analysis, both univariate and multivariate models were adopted, with adjustments for age at the time of diagnosis and various cancer clinicopathological features. These models enabled the calculation of odds ratios along with their corresponding 95% confidence intervals (CIs) and *p*-values, using logistic regression. The analysis of differences in PFS and OS involved the performing of hazard ratios (HRs) derived from univariate and multivariate Cox proportional-hazard models. The survival curves were constructed and assessed employing the log-rank test, and the Kaplan–Meier method was used for generating these curves. Throughout the entirety of the analysis, a *p*-value less than 0.05 was deemed statistically significant.

## 3. Results

### 3.1. Clinical Characteristics

**Chart 1:**
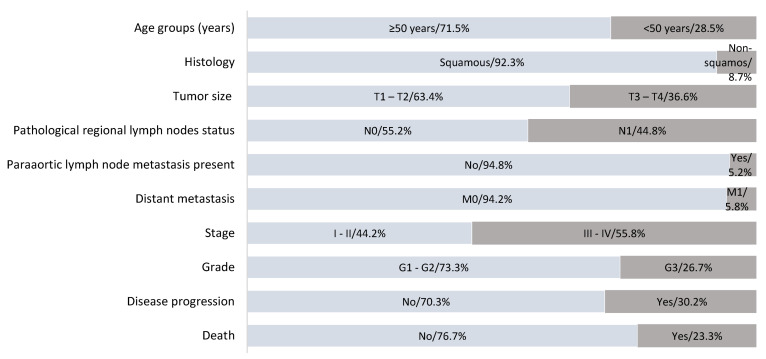
Patient and tumor data from 172 study participants (variables/subgroups/frequencies (%)). T1 + T2—smaller tumor size; T3 + T4—larger tumor size; N0—no regional lymph node metastasis; N1—positive regional lymph node metastasis; M0—no distant metastasis; M1—positive distant metastasis; G1–G2—well and moderately differentiated tumor; G3—poorly differentiated tumor, Stages I–II—lower stages; Stages III–IV—advanced stages.

In the course of our investigation, the study cohort primarily comprised Lithuanian nationals, constituting 90.1% of the participants, with the remaining individuals originating from other European countries. The demographic profile of the subjects exhibited a broad spectrum of ages, spanning a considerable range from 22 to 83 years. When the participants were diagnosed, their mean age stood at 55.4 years, with a standard deviation of 13.5 years, indicating the spectrum of ages represented in this study. An in-depth analysis of the tumor size dimensions unveiled a noteworthy predominance of the T2 category, constituting 48.8% of the cases. Lymph node involvement was documented in 44.8% of the patient cohort. Furthermore, the study uncovered that metastasis to paraaortic lymph nodes was documented in 5.2% of the cases. Distant metastasis was detected in 10 cases, constituting 5.8% of the total. Cancer staging indicated that IIB and IIIC1 were the prevailing stages, representing 32% and 31% of the cases, respectively. This stratification also revealed that lower stages (I–II) accounted for 44.2% of the participants, while the more advanced stages (III–IV) encompassed 55.8% of the study population. Further scrutiny unveiled a distribution of tumor differentiation, with 7.6% classified as well-differentiated (G1), 65.7% as intermediate (G2), and 26.7% as poorly differentiated, thereby illustrating the heterogeneity of tumor grades within the study cohort. Regarding treatment, a significant majority of patients, amounting to 69.2%, underwent standard chemoradiation therapy. The remaining participants underwent surgery followed by radiotherapy or systemic treatment. Importantly, a substantial 70.3% of the patients exhibited a complete response to treatment, while 21.5% showed a partial response. A smaller segment, comprising 8.2%, exhibited either stable disease or progressive disease. Within the context of progression, the median progression-free survival (PFS) was calculated at 13 months, exhibiting a range spanning from a minimum of 1 month to a maximum of 201 months. Over the course of the follow-up period, disease progression was confirmed in 52 cases, impacting 30.2% of the cohort. A substantial majority of those experiencing progression exhibited localized advancement and metastasis in regional lymph nodes, affecting 51 patients, while an additional 18 cases demonstrated progression in paraaortic lymph nodes. The disease also metastasized in 16 patients. Regrettably, 40 events of death occurred during the follow-up period, accounting for 23.3% of the cohort. The median overall survival (OS) spanned from 1 to 201 months, with the midpoint recorded at 16.5 months. Notably, 45.9% of the patients had concurrent chronic diseases, yet the underlying cause of death in all cases was the relentless progression of cancer. Chart 1 offers a comprehensive breakdown of clinicopathological features.

### 3.2. SNP Frequencies

In our study, a total of 172 patients underwent genotyping for a set of five *RRP1* SNPs: rs2838342, rs7276633, rs2051407, rs9306160, and rs762400. Among these, rs9306160 was identified in 169 cases, with three cases excluded due to non-amplification. It is noteworthy that all of the SNPs examined were found to be in accordance with the Hardy–Weinberg equilibrium, as indicated by *p*-values exceeding 0.05. Upon comparing the allele frequencies determined within our cohort to those of the European population data from the 1000 Genomes project, we detected slight yet statistically significant disparities in the minor allele frequencies (MAF) for all of the SNPs, with *p*-values > 0.05. Comprehensive details regarding genotype and allele frequencies can be found in Table 2.

### 3.3. Linkage Disequilibrium and Haplotypes Distribution

**Figure 1:**
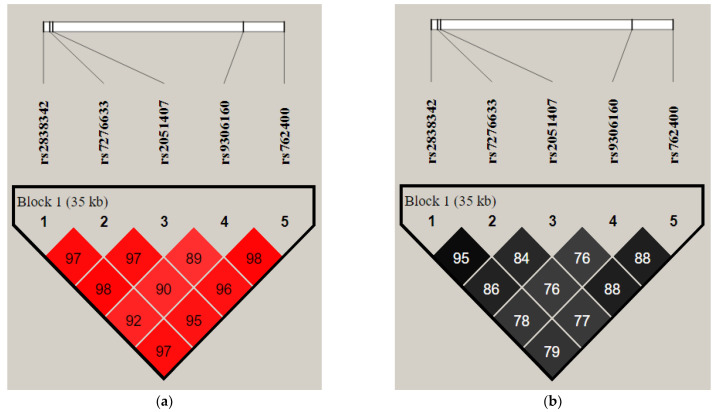
The LD data for *RRP1B* single-nucleotide polymorphisms include numerical values and color coding for both r-squared and D′, providing insights into the linkage disequilibrium. Pairwise linkage disequilibrium (LD) pattern for *RRP1B* polymorphisms in cervical cancer patients. (**a**) The color LD plot indicates the strength of D′, with dark red representing strong LD. (**b**) The color LD plot indicates the strength of *r*^2^, with dark grey representing strong LD.

In our analysis of linkage disequilibrium (LD) among the SNPs in the *RRP1B* gene, we calculated two commonly used measures: D’ and r^2^ (Figure 1). For D’, the mean value was approximately 0.949 ± 0.037, indicating a relatively strong LD on average. The range of D’ values varied from a minimum of 0.907 to a maximum of 0.987. Similarly, for r^2^, the mean value was approximately 0.802 ± 0.065, suggesting a moderate-to-high degree of LD on average. The range of r^2^ values spanned from a minimum of 0.761 to a maximum of 0.953. These findings provide insights into the patterns of LD within the RRP1B gene, highlighting regions of potential genetic linkage and association. Based on the calculated mean values and the range of D’ and r^2^ values, it appears that there is a significant level of linkage disequilibrium (LD) among the SNPs in the *RRP1B* gene. The mean values for both D’ and linkage disequilibrium r^2^ indicate a relatively strong LD on average, and the range of values suggests consistency in LD across the analyzed SNPs. Therefore, it would be reasonable to conclude that LD between these SNPs in the *RRP1B* gene is indeed strong. Due to the observed strong linkage disequilibrium (LD) among the SNPs within the *RRP1B* gene, it was decided to include all five SNPs in haplotype analysis. This decision was based on the premise that SNPs in strong LD tend to be inherited together as haplotype blocks, allowing for a more comprehensive understanding of the genetic variations within this genomic region. By analyzing haplotypes constructed from these SNPs, we aimed to capture the collective influence of genetic variations on phenotypic traits or disease susceptibility, thereby enhancing the depth of our genetic investigation.

The results revealed a variety of haplotypes present among the tested individuals. Thirteen haplotypes were identified (Table 3). Among the identified haplotypes, the most prevalent was “ATCCG”, accounting for approximately 55% of the total haplotypes observed. Following closely behind, “GCTTC” constituted around 36% of the haplotypes. Other haplotypes, such as “ATCTG”, “ATCTC”, “GCCTG”, etc., were observed at lower frequencies, each comprising less than 10% of the total haplotypes. The diversity in haplotype composition suggests genetic variability within the *RRP1B* gene region among the studied population. Understanding the distribution of these haplotypes can provide valuable insights into genetic susceptibility, disease association, and population genetics within the context of our research objectives.

For further analysis of associations, we focused on the two most common haplotypes observed in our study, namely “ATCCG” and “GCTTC”.

### 3.4. Association Analysis

All the examined polymorphisms exhibited statistically significant associations with the clinical manifestations of cervical cancer. However, we did not find any statistically significant associations between SNPs and nodal involvement or tumor differentiation. On the other hand, all the polymorphisms were linked to tumor size or metastasis. Furthermore, some of them appeared to influence cancer stage and prognosis. The tabulated results furnish us with a trove of statistical insights. This meticulous analysis unveils the intriguing associations between specific SNPs and an array of vital tumor characteristics, offering a multifaceted perspective on the clinical attributes of cervical.

#### 3.4.1. Rs2838342

The analysis of SNP rs2838342 yielded noteworthy results. According to the univariate logistic regression analysis, individuals with the presence of the A allele (A allele +) exhibited a significantly lower odds ratio (OR) of 0.281 (95% CI: 0.122–0.643, *p* = 0.002) for advanced tumor size (T3–T4) when compared to those with its absence (A −), indicating a significantly reduced likelihood of advanced tumor size (T3–T4). In the multivariate logistic regression analyses across four models, the A allele was consistently associated with significantly lower odds of larger tumor size, with an OR of 0.280 (95% CI: 0.122–0.643, *p* = 0.003) in the presence of patient age at diagnosis (Model No. 1). Model No. 2 introduced additional covariates (age at diagnosis and tumor differentiation grade) and continued to demonstrate a consistent association between rs2838342 and the tumor size (OR = 0.299, 95% CI: 0.129–0.692, *p* = 0.005). However, a possible trend emerged where G3 was associated with higher odds of larger tumor size (OR = 1.991, 95% CI: 0.978–4.051, *p* = 0.058). Model No. 3 expanded the analysis to include the presence of regional lymph node involvement (N1 versus N0). In this model, rs2838342 remained associated with tumor size (OR = 0.244, 95% CI: 0.096–0.619, *p* = 0.003), but the addition of N1 as a covariate substantially increased the odds of larger tumor size (OR = 7.367, 95% CI: 3.347–16.217, *p* < 0.001). Model No. 4 further extended the analysis to consider the presence of distant metastasis (M1 versus M0), further supporting a significant relationship (OR = 0.266, 95% CI: 0.102–0.691, *p* = 0.007). Throughout all these models, the association between rs2838342 and tumor size persisted. In summary, these multivariate logistic regression analyses, while adjusting for covariates, reveal a robust and consistent association between the presence of at least one A allele and a lower risk of larger tumor size. In genotypic models, the GG genotype showed an increased odds ratio of 2.160 (95% CI: 0.867–5.380). Nevertheless, this difference did not reach statistical significance (*p* = 0.098).

The univariate logistic regression analysis revealed that the A allele significantly reduced the odds of having distant metastasis (OR = 0.274, 95% CI: 0.072–1.040, *p* = 0.044). Conversely, when comparing the presence of at least one G allele to having none (G allele + vs. G −), the OR was 1.199, indicating slightly higher odds of having distant metastasis. However, this association was not statistically significant (*p* = 0.798). Across all multivariate models for the A allele + versus A − comparison, when adjusting for age, G, N, and tumor size, the OR suggests a potential protective effect of the A allele in reducing the risk of distant metastasis. Unfortunately, statistical significance was limited (all cases *p*-value > 0.05). These results provide preliminary evidence that the A allele of rs2838342 might play a protective role against the development of distant metastasis.

In the analysis, focused on a worse prognosis group, characterized by T3–T4 tumor stages and the G3 tumor grade, the A allele of rs2838342 significantly reduces the likelihood of a worse prognosis (T3–T4 + G3) compared to those with its absence (A −), with an OR of 0.182. The 95% CI spans from 0.061 to 0.538, and the *p*-value is a strikingly low 0.002. The GG genotype of rs2838342 presents a notably high OR (3.000) for a worse prognosis (T3–T4 + G3) when compared to the AA genotype. Although the *p*-value (0.071) suggests a potential association, it did not reach conventional significance levels. Similarly, the G allele of rs2838342 does not significantly impact the likelihood of a worse prognosis, as reflected in the wide 95% CI from 0.274 to 1.847 and a *p*-value of 0.485.

While the genotypes did not show a significant association in the comparison of positive Stages III–IV versus Stages I–II, allelic comparisons provided additional insights. The A allele demonstrated a substantially lower odds ratio (OR = 0.341, 95% CI = 0.137–0.849, *p*-value = 0.017), suggesting a potential protective effect in the context of advanced cancer stages. Conversely, the G allele did not exhibit a statistically significant association (*p*-value = 0.239).

When considering age as a dichotomous variable (≤50 vs. >50 years), those carrying the AG genotype had a reduced risk of developing cervical cancer before the age of 50 (OR = 0.471, 95% CI: 0.226–0.983, *p* = 0.045).

These findings underscore the potential relevance of the rs2838342 SNP in influencing the progression and severity of cervical cancer, particularly in the transition from early to advanced stages. The protective effect associated with specific genotypes and alleles implies a potential role for rs2838342 as a prognostic marker in cervical cancer patients. All the results are presented in Table 4 and Table 5.

#### 3.4.2. Rs7276633

The carriers of the T allele in rs7276633 were significantly associated with a decreased risk of falling into the higher tumor size category (T3–T4), with an odds ratio (OR) of 0.281 (95% CI = 0.122–0.643, *p* = 0.003). Moving on to the multivariate logistic regression analysis, the findings remain consistent across all four models. In multivariate Model No.1, the presence of the T allele (+) is significantly associated with a reduced risk of having a higher tumor size compared to the absence of the T allele (−). This association is statistically significant with an odds ratio (OR) of 0.280 and a 95% confidence interval (CI) of 0.122–0.643 (*p* = 0.003). This association persists in Model No. 2, demonstrating a significant reduction in the odds of higher tumor size (OR = 0.299, 95% CI: 0.129–0.692, *p* = 0.005). Models Nos.3 and 4 also support this finding, with ORs of 0.277 and 0.264 (95% CI: 0.125–0.708, *p* = 0.003; 95% CI: 0.147–0.893, *p* = 0.007, respectively). Conversely, the C allele of rs7276633 did not exhibit a significant association with tumor size (*p* = 0.145). Otherwise, the trend of the CC genotype compared to the TT genotype showing an increased risk of higher tumor size remained consistent across all models, although this association was non-significant.

Patients with the presence of the T allele (+) were significantly associated with a reduced risk of higher tumor stage (III–IV) (OR = 0.341, 95% CI: 0.137–0.849, *p* = 0.021) and worse prognosis (T3–T4 + G3) (OR = 0.182, 95% CI: 0.061–0.538, *p* = 0.002), while the C allele did not exhibit significant associations with the parameters studied. In conclusion, these findings imply that the T allele of rs7276633 might confer a protective effect against advanced tumor size and prognosis. Moreover, carriers of the C allele were at a higher risk of developing the disease at an age younger than 50 years (OR = 2.138, 95% CI: 1.080–4.230, *p* = 0.029). The results are presented in Table 5 and Table 6.

#### 3.4.3. Rs2051407

There were no significant associations between genotypes and clinicopathomorphological features. However, the presence of the C allele (+) was associated with a decreased risk of having a larger tumor (T3–T4) compared to those without the C allele (C −) (OR 0.393, 95% CI of 0.166–0.929, *p* = 0.033). This association was consistent and statistically significant across three multivariate analysis models, when the adjustment of age and tumor clinicopthatological features was made (Model No. 1: OR 0.392, 95% CI: 0.166–0.928, *p* = 0.033; Model No. 2: OR 0.414, 95% CI: 0.173–0.992, *p* = 0.048; Model No. 3: OR 0.354, 95% CI: 0.134–0.930, *p* = 0.035, respectively). But Model No.4 shows that the association did not reach statistical significance (OR 0.409, 95% CI: 0.149–1.123, *p* = 0.083).

In the univariate logistic regression analysis, investigating the association between alleles and metastasis, carrying the C allele significantly decreased the chance of having metastasis, with an OR of 0.223 (95% CI: 0.058–0.858) and a *p*-value of 0.019. These findings suggest that the presence of the C allele may serve as a protective factor against metastasis. In multivariate analysis, Models Nos. 1, 2, and 3, showed a consistent association between the presence of the C allele and a reduced risk of metastasis (*p* = 0.030, *p* = 0.038, *p* = 0.037, respectively). However, Model No. 4 did not yield significant results for this polymorphism, when the adjustment of age, G, N, and T was made. Tumor stage (T3–T4) was consistently identified as a significant predictor of metastasis in all models.

The presence of the C allele was associated with a significantly reduced risk of transitioning to a worse prognosis disease (T3–T4 + G3), as evidenced by an OR of 0.267 (95% CI: 0.087–0.823, *p* = 0.021), suggesting that it may serve as a protective factor. These findings highlight the potential influence of this SNP on the expected prognosis of the disease. The results are presented in Table 5 and Table 7.

#### 3.4.4. Rs9306160

The analysis suggests that the rs9306160 SNP may have a significant protective effect against metastasis, as indicated by the statistically significant result for the C allele (+) (OR = 0.179, 95% CI: 0.044–0.721, *p* = 0.008). There was no significant association between the CT genotype and the presence of metastasis. However, for the TT genotype compared to CC, the OR was 5.889 (95% CI: 0.993–34.906) with a *p*-value close to the significance threshold at 0.051. This implies a potential trend towards an increased risk of metastasis for the TT genotype. In the multivariate logistic regression analysis for metastasis (M), Model No. 1 showed that the presence of the C allele (+) significantly reduced risk of metastasis (OR = 0.187, 95% CI: 0.046–0.760, *p* = 0.019). Model No. 2 continued to show a protective effect the C allele (+) with an OR of 0.166 (95% CI: 0.039–0.702, *p* = 0.015). In Model No. 3 the C allele (+) still exhibited a protective effect (OR = 0.151, 95% CI: 0.032–0.717, *p* = 0.017). This confirms the significantly reduced risk of metastasis associated with the C allele. However, in Model No.4, the protective effect is not statistically significant, while there is a protective trend. The results are presented in Table 8.

#### 3.4.5. Rs762400

This SNP also showed significant results. The G allele was significant for a reduced risk of advanced tumor size (T3–T4) compared to the absence of the G allele (G −) (OR = 0.383, 95% CI: 0.151–0.967, *p* = 0.037). Based on multivariate logistic regression analysis, taking into account age, tumor grade, nodal involvement, and distant metastasis, the association maitains significance in the initial models: Model No. 1 (OR = 0.383, 95% CI: 0.151–0.968, *p* = 0.042), Model No. 2 (OR = 0.378, 95% CI: 0.148–0.970, *p* = 0.043), and Model No. 3 (OR = 0.330, 95% CI: 0.115–0.946, *p* = 0.039). However, in Model No. 4, the association was not statistically significant (*p* = 0.106). Thus, the results indicated that the role of other covariates is more important with regard to the impact of the G allele.

Moreover, the univariate logistic regression suggests that individuals carrying the G allele (+) had a significantly lower risk of having metastasis (OR = 0.176, 95% CI: 0.045–0.686, *p* = 0.006). The multivariate analyses reinforce this association, with the presence of the G allele consistently linked to a reduced risk of metastasis. This significance holds in Models Nos. 1, 2, and 3 (OR = 0.165, 95% CI: 0.042–0.659, *p* = 0.011; OR = 0.168, 95% CI: 0.042–0.673, *p* = 0.012; OR = 0.149, 95% CI: 0.032–0.703, *p* = 0.016, respectively). In Model No. 4, while the association between the G allele and metastasis does not reach conventional statistical significance, it still suggests a notable trend towards a reduced risk of metastasis associated with the G allele. Importantly, age and other clinical factors did not demonstrate significant associations with metastasis (Table 9).

#### 3.4.6. Haplotypes

With the understanding that rs2838342, rs7276633, rs2051407, rs9306160, and rs762400 may not act independently, we opt to explore haplotypes. By analyzing haplotypes, we aim to capture the combined effect of multiple SNPs within the gene, thus providing a more comprehensive understanding of the genetic landscape and its potential implications in our study. We meticulously analyzed the associations between diplotypes and various clinical characteristics. Specifically, we examined the heterozygous diplotype (ATCCG/alternative haplotype) versus the homozygous diplotype (ATCCG/ATCCG), ATCCG haplotype non-carriers versus the homozygous diplotype (ATCCG/ATCCG), heterozygous diplotype (GCTTC/alternative haplotype) versus the homozygous diplotype (GCTTC/GCTTC), GCTTC haplotype non-carriers versus the homozygous diplotype (GCTTC/GCTTC), and heterozygous diplotype (ATCCG/GCTTC) carriers versus non-carriers.

Significantly, GCTTC haplotype non-carriers exhibited a greater protective effect against advanced tumor size (T3–T4) and metastasis compared to those with the homozygous diplotype (GCTTC/GCTTC) (OR = 0.367, 95% CI: 0.136–0.992, *p* = 0.038; OR = 0.098, 95% CI: 0.016–0.578, *p* = 0.010, respectively). This finding suggests a potential role of genetic variations represented by the GCTTC haplotype in promoting aggressive tumor behavior. For patients with advanced tumor stages (III–IV versus I–II) and worse prognosis (T3–T4 + G3 versus T1–T2 + G1–G2), individuals lacking the ATCCG haplotype showed a significantly higher likelihood of exhibiting advanced tumor stages and being in the worse prognosis group compared to those with the homozygous diplotype (ATCCG/ATCCG) (OR = 1.250, 95% CI: 0.454–3.444, *p* = 0.032; OR = 2.100, 95% CI: 0.638–6.916, *p* = 0.048, respectively) (Table 10).

Table 11 presents the results of multivariate logistic regression analyses focusing on diplotypes, with adjustments made for clinicopathological characteristics, particularly emphasizing tumor size and metastasis. Model 1: In the initial model, we adjusted for age (years) as an additional covariate. The association between GCTTC haplotype non-carriers and reduced odds of advanced tumor size remained significant (OR = 0.393, 95% CI: 0.188–0.822, *p* = 0.039). This underscores the robustness of our initial findings, indicating that age did not substantially alter the observed relationship between haplotype status and tumor size. Model 2: Further adjustments were made by including tumor grade (G3 versus G1 + G2) in the analysis. Despite this additional adjustment, the association between GCTTC haplotype non-carriers and decreased odds of advanced tumor size remained statistically significant (OR = 0.392, 95% CI: 0.185–0.827, *p* = 0.041). This suggests that the observed association is independent of tumor grade, emphasizing the potential importance of genetic factors in influencing tumor progression. Model 3: Despite the inclusion of nodal status in the analysis, the association between GCTTC haplotype non-carriers and reduced odds of advanced tumor size remained statistically significant (OR = 0.391, 95% CI: 0.173–0.884, *p* = 0.041). Model 4: Finally, we included metastasis (M1 versus M0) as an additional covariate in the analysis. The association between GCTTC haplotype non-carriers and advanced tumor size showed a trend towards significance (OR = 0.380, 95% CI: 0.166–0.869, *p* = 0.046). On the focus on metastasis, in Model No.1, GCTTC haplotype non-carriers exhibit a substantial protective effect against metastasis (OR = 0.101, 95% CI 0.017–0.598, *p* = 0.012). This suggests a potential role of genetic variations represented by the GCTTC haplotype in influencing metastatic propensity, even after adjusting for age. In Model No. 2, which includes additional adjustments for tumor grade (G3 versus G1 + G2), the protective effect against metastasis remains significant (OR = 0.095, 95% CI 0.016–0.577, *p* = 0.011), further emphasizing the independent nature of this association. Model No.3 incorporates adjustments for lymph node involvement (N1 versus N0) along with age and tumor grade. Despite these additional adjustments, the protective effect against metastasis among GCTTC haplotype non-carriers persists (OR = 0.075, 95% CI 0.011–0.534, *p* = 0.010), highlighting the robustness of the observed association. Finally, in Model No.4, which includes adjustments for tumor stage (T3–T4), in addition to age, tumor grade, and lymph node involvement, the protective effect against metastasis remains significant (OR = 0.150, 95% CI 0.023–0.965, *p* = 0.048). This suggests that the influence of genetic variations represented by the GCTTC haplotype on metastatic propensity is independent of tumor size and other clinicopathological factors.

Overall, the consistent significance of the protective effect across all models underscores the potential importance of genetic variations represented by diplotypes in predicting tumor size and metastasis in cervical cancer patients, irrespective of traditional clinicopathological factors.

### 3.5. Survival Analysis

The influence of the SNPs on survival, both progression-free survival (PFS) and overall survival (OS), was assessed using genotype and allelic models. In Cox’s univariate and multivariate models for PFS and OS, we assessed the impact of SNPs on survival outcomes. No significant link between SNP‘s genotypes or alleles and PFS was detected. In the case of SNP Rs9306160, the survival analysis did not yield differences for the genotypes and alleles.

But the effect of four SNPs (rs2838342, rs7276633, rs2051407, rs762400) on OS has been identified as important.

The results indicate that for SNP rs2838342, there were no statistically significant associations between genotypes and survival outcomes. However, the presence of the A allele displayed a considerably lower hazard, signifying a potential protective role. This observation is particularly noteworthy, as the *p*-value of 0.031 indicates that the A allele may significantly contribute to improved OS outcomes (HR = 0.465, 95% CI: 0.232–0.931). Utilizing Cox’s multivariate models, an effect of the A allele was sustained even after adjusting for age at diagnosis (HR = 0.462, 95% CI: 0.231–0.926, *p* = 0.030, Model No. 1). When scrutinizing the influence of age at diagnosis and broader tumor characteristics (tumor T, N, G) in Model No. 2, we observed that the impact of the A allele on OS has now become statistically insignificant. We must note that the effect of tumor size was significant on the survival outcome, revealing an HR of 7.463 (T3–T4 vs. T1–T2, *p* < 0.001), reflecting its substantial impact on OS.

Similarly, for SNP rs7276633, the TC and CC genotypes did not show significant differences in survival when compared to TT, but the presence of the T allele was associated with better OS (HR = 0.465, CI: 0.232–0.931, *p* = 0.031). The T allele’s protective effect persisted, with an adjusted HR of 0.462 (95% CI: 0.231–0.926, *p* = 0.030) after accounting for age-related factors (Model No. 1). These findings underscore the significance of allelic effects in influencing overall survival. Regrettably, Model No. 2 did not produce statistically significant results when adjusting for tumor T, N, G, and patients’ age. The impact of tumor size on OS was significant, with an HR of 7.463 (95% CI: 3.195–17.432, *p* < 0.001).

Next, our attention turned to SNP rs2051407. Like previous SNPs, the different genotypes showed no substantial differences in survival outcomes. However, the C allele was a factor in modulating Patient OS. The C allele carriers had a decreased risk of dying faster (HR = 0.418, CI: 0.204–0.858, *p* = 0.017). In multivariate Cox‘s regression analysis, the C allele remains a factor for longer overall survival (HR = 0.404, 95% CI: 0.196–0.832, *p* = 0.014), when adjusting for the age of patients (Model No. 1). Unfortunately, Model No. 2 did not yield statistically significant results, with a significant effect of tumor size on overall survival persisting (HR = 7.484, 95% CI: 3.227–17.355, *p* < 0.001).

Finally, our analysis extended to SNP rs762400. In the univariate model, patients with the CC genotype, compared to the GG genotype, exhibited a significant impact on OS, with an HR of 2.550 (95% CI: 1.098–5.923, *p* = 0.030), indicating an elevated risk of adverse outcomes for individuals carrying this genotype. This result remained significant in multivariate Model No.1, controlling for patient age (HR = 2.476, 95% CI: 1.064–5.758, *p* = 0.035). Exploring the interplay of tumor characteristics and age at diagnosis in Model No. 2, advanced tumor size (T3–T4 versus T1–T2) once again emerged as a significant predictor, displaying a substantial HR of 7.546 (95% CI: 3.250–17.520, *p* < 0.001). In this model, the CC genotype still increases the risk for shorter OS, but the significance level (*p*) is >0.05. Moreover, the scrutiny of SNP rs762400 showcased that the allelic model does not contradict the results of the genotypic model. The presence of the G allele emerged as a significant protective factor. The holders of G allele were less likely to have shorter OS when compared to the non-carriers (HR = 0.374, CI: 0.177–0.788, *p* = 0.010). The presence of the G allele (+) was associated with an HR of 0.370 (95% CI: 0.176–0.781, *p* = 0.009) in Model No. 1, after adjusting for age, indicating a substantially reduced risk of adverse OS outcomes linked to this genetic variant. In multivariate Model No. 2, advanced tumor size (T3–T4) exhibited a significant HR of 7.496 (95% CI: 3.235–17.373, *p* < 0.001). These results underline the considerable impact of tumor characteristics on OS outcomes, and once again, the significant influence of the G allele for OS was not observed.

In our analysis, using Cox’s univariate model for progression-free survival (PFS) and overall survival (OS), we observed interesting trends in the association between *RRP1B* haplotypes and patient outcomes. Specifically, the ATCCG haplotype non-carriers versus the homozygous diplotype (ATCCG/ATCCG) showed an elevated hazard ratio (HR) for both PFS and OS, indicating a potential link between this haplotype and poorer survival outcomes. However, statistical significance was not achieved in this comparison.

Conversely, the heterozygous diplotype of GCTTC/alternative haplotype, compared to the homozygous diplotype (GCTTC/GCTTC), displayed a significantly decreased HR for OS (HR = 0.274, 95% CI: 0.120–0.626, *p* = 0.002), suggesting a possible protective effect associated with this haplotype. Similarly, GCTTC haplotype non-carriers compared to the homozygous diplotype (GCTTC/GCTTC) also exhibited a significantly decreased HR for OS (HR = 0.298, 95% CI: 0.128–0.695, *p* = 0.005), indicating a potentially favorable impact on survival outcomes.

In our comprehensive analysis using Cox’s multivariate models for overall survival (OS), we meticulously examined the adjusted associations between diplotypes, age at diagnosis, and various tumor characteristics. Focusing on diplotypes, particularly the comparison between the heterozygous diplotype (GCTTC/alternative hap) and the homozygous diplotype (GCTTC/GCTTC), our findings consistently demonstrated significantly decreased odds of overall survival (OS) across both Model Nos. 1 and 2 (OR = 0.259, 95% CI: 0.113–0.597, *p* = 0.002; OR = 0.372, 95% CI: 0.153–0.904, *p* = 0.029, respectively). This suggests a potential protective effect associated with certain diplotypes, indicating their relevance as prognostic indicators in cervical cancer. Similarly, when comparing GCTTC haplotype non-carriers to the homozygous diplotype (GCTTC/GCTTC), we observed notably reduced odds of OS in both Model Nos. 1 and 2 (OR = 0.303, 95% CI: 0.130–0.708, *p* = 0.006; OR = 0.363, 95% CI: 0.151–0.871, *p* = 0.023, respectively). This reinforces the importance of haplotype status in predicting survival outcomes, further highlighting the potential clinical significance of genetic variations represented by diplotypes.

In summary, our multivariate analysis within the Cox regression framework unraveled the intricate relationships between genetic variations, age at diagnosis, and tumor characteristics, providing a nuanced understanding of their combined impact on overall survival in this particular context. Tumor characteristics played a significant role, unveiling HRs and reflecting their substantial impact on OS.

**Figure 2:**
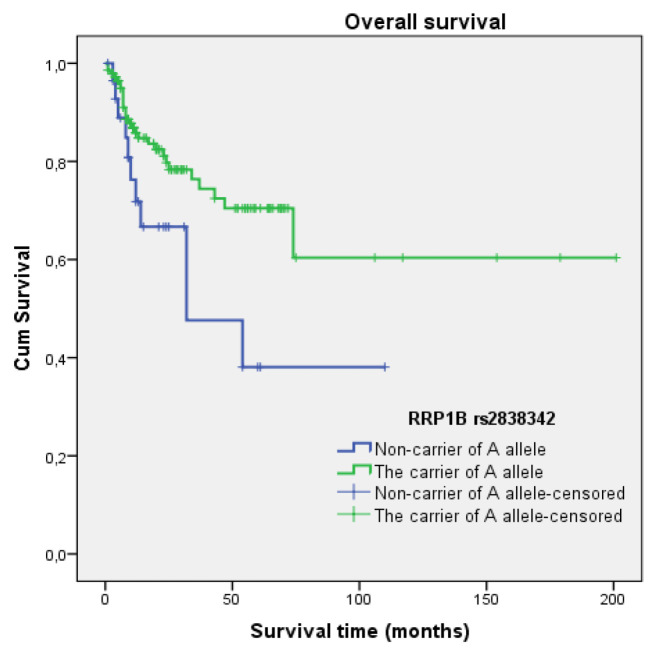
Kaplan–Meier survival curves were generated for patients with cervical cancer based on the rs2838342 polymorphism in the allelic model, illustrating differences in OS (*n* = 172). The *y*-axis displays the probability of survival, while the *x*-axis represents the duration in months from the diagnosis of cervical cancer, verifying the occurrence date of the event of interest (OS). Each vertical step in the curve signifies events (i.e., deaths), and right-censored patients are denoted by a vertical mark in the curve at the censoring time. Carrying the A allele of rs2838342 was associated with an increased likelihood of longer overall survival (HR = 0.465, 95% CI: 0.232–0.931, *p* = 0.031).

**Figure 3:**
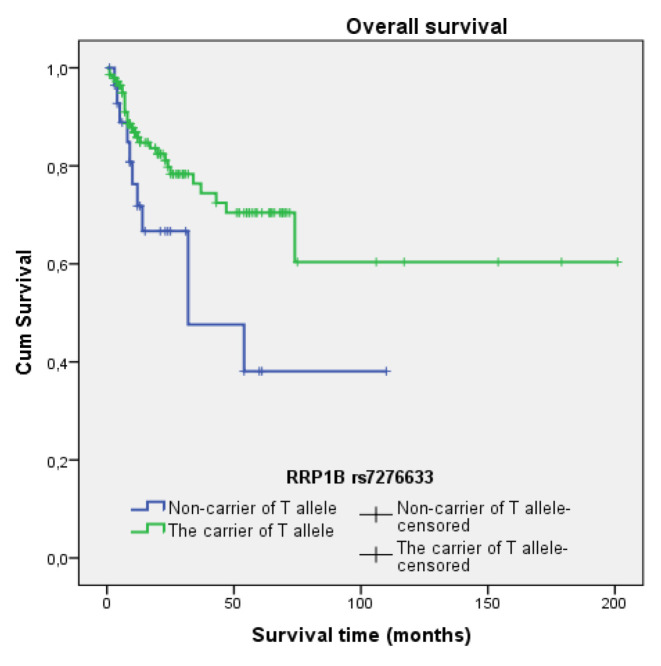
Kaplan–Meier survival curves for OS in patients with cervical cancer, according to rs7276633 polymorphism (*n* = 169). Carrying the T allele in rs7276633 increased the possibility for longer OS (HR = 0.465, CI: 0.232–0.931, *p* = 0.031).

**Figure 4:**
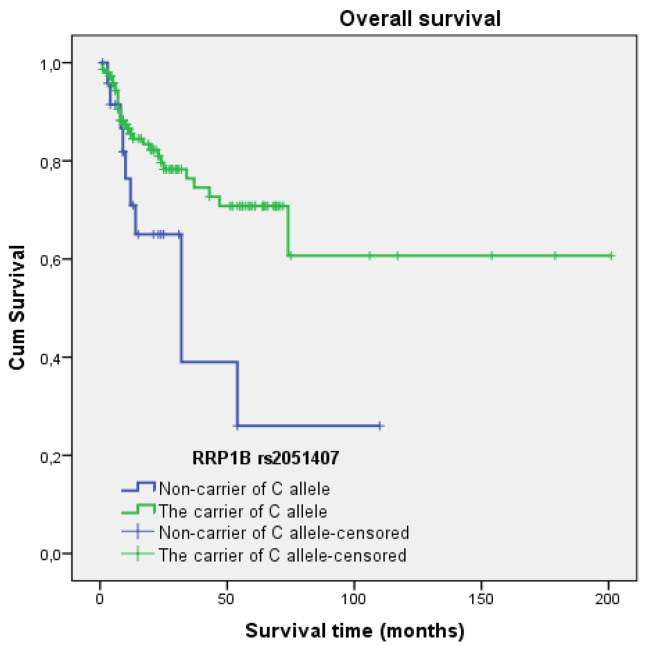
Kaplan–Meier survival curves for OS in patients with cervical cancer, according to rs2051407 polymorphism (*n* = 172). Carriers of the rs2051407 C allele had an increased chance of longer OS in comparison with non-carriers (HR = 0.418, CI: 0.204–0.858, *p* = 0.017).

**Figure 5:**
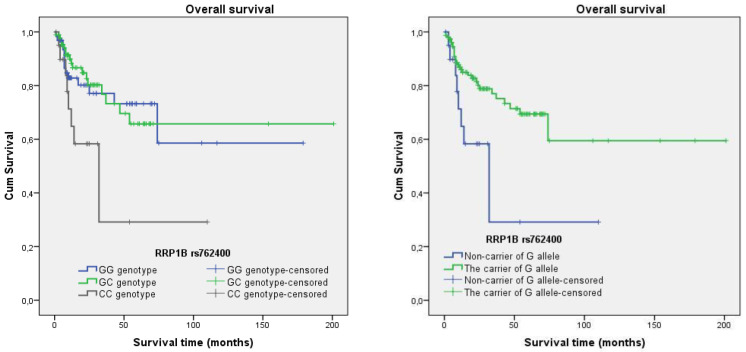
Kaplan–Meier survival curves for OS in patients with cervical cancer, according to rs762400 polymorphism (*n* = 172). Rs762400 CC genotype increased the risk for shorter OS compared to patients with the GG genotype (HR = 2.550, 95% CI: 1.098–5.923, *p* = 0.030). Individuals carrying the G allele exhibited a heightened likelihood of longer OS compared to those without the G allele (HR = 0.374, CI: 0.177–0.788, *p* = 0.010).

**Figure 6:**
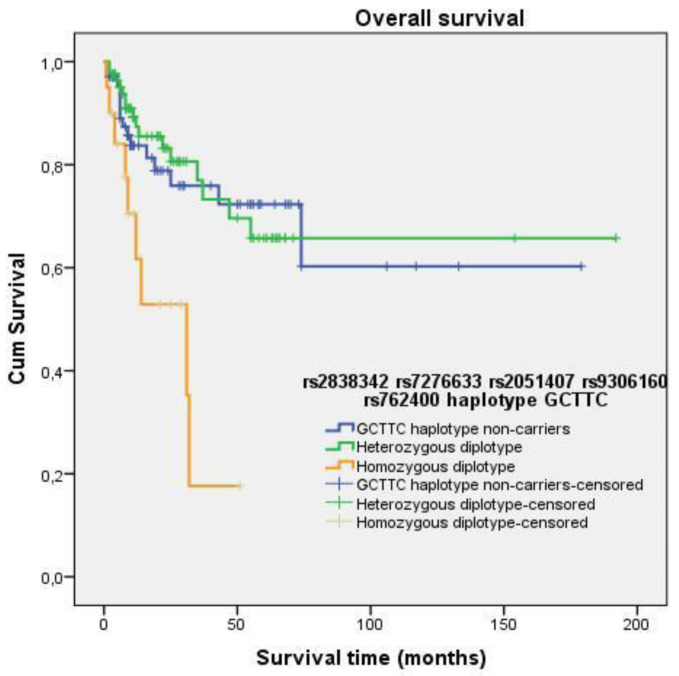
Kaplan–Meier survival curves for OS in patients with cervical cancer stratified by diplotypes at rs2838342, rs7276633, rs2051407, rs9306160, and rs762400, with a focus on the GCTTC haplotype (*n* = 172). The heterozygous diplotype (GCTTC/alternative haplotype), compared to the homozygous diplotype (GCTTC/GCTTC), displayed a significantly decreased hazard ratio (HR = 0.274, 95% CI: 0.120–0.626, *p* = 0.002), suggesting a possible protective effect associated with this haplotype. Similarly, GCTTC haplotype non-carriers compared to the homozygous diplotype (GCTTC/GCTTC) also exhibited a significantly decreased hazard ratio for OS (HR = 0.298, 95% CI: 0.128–0.695, *p* = 0.005).

All the results are presented in Table 12, Table 13, Table 14 and Table 15. Kaplan–Meier analysis was performed to generate survival curves for genotypes, alleles, and haplotypes showing significant associations with overall survival (OS) (Figure 2, Figure 3, Figure 4, Figure 5 and Figure 6).

## 4. Discussion

Despite prevention programs and vaccination efforts, cervical cancer continues to pose a significant global public health challenge. Mortality rates vary across different regions of the world, with the majority of deaths occurring in low- and middle-income countries [27,33]. With various risk factors such as human papillomavirus, sexual activity, oral contraceptives, immunosuppression, family history, and various molecular factors (including *HOX*, *PI3K/AKT/mTOR*, *EGFR*, *PDGFR*, *VEGF* genes) influencing cervical cancer progression and metastasis [34], a comprehensive understanding of genome variations and biological characteristics, set against the backdrop of environmental modifications, will enhance the accuracy of disease diagnosis and treatment. This knowledge will pave the way for more precise, personalized, and effective therapeutic protocols tailored to individual patients.

In our study, all examined SNPs exhibited significant associations with clinicopathological features of cervical cancer. Rs2838342, rs2051407, and rs762400 were linked to tumor size (T) and metastasis (M), while rs7276633 was associated with tumor size and rs9306160 was associated with metastasis. When analyzing the prognosis of the disease, considering tumor size and differentiation, significant results were observed in cases involving rs2838342, rs7276633, and rs2051407. Additionally, rs2838342 and rs7276633 were associated with the stage of the disease and patients’ age groups. Based on these abundant and trending findings, it can be anticipated that *RRP1B* SNPs play a role in influencing the aggressiveness of cervical cancer and and the risk of metastasis.

Regrettably, our data could not be compared with that of other researchers, as we were unable to find publications specifically investigating and analyzing *RRP1B* polymorphisms in cervical cancer cases. Evaluating the results of rs2838342, rs7276633, rs2051407, and rs762400 polymorphisms poses particular difficulties. In some cases, explaining the lack of correspondence between the genotypic model and the allelic model in the associations with the clinical characteristics of the tumor is challenging, especially due to the absence of published results from studies analyzing these SNPs. The analysis of these four polymorphisms clearly delineated the tendency of the more common allele to enhance overall survival. However, further replication of these findings is still needed.

A review of the global literature focused on the expression levels of *RRP1B*.

Crawford et al. conducted research on breast cancer. Expression of *RRP1B*, and the activity of *RRP1B* expression, was investigated to be higher in low-metastatic mice inbred strains with mammary cancer compared to high-metastatic strains. Additionally, the variation in *RRP1B* expression within a highly metastatic mouse mammary tumor cell line was found to modify progression. Ectopic Expression of *RRP1B* reduced tumor growth and metastatic potential. Expression of this gene also predicted survival in human breast cancer. A significant difference in overall survival for the groups with good and poor prognosis, predicted by the *RRP1B* activation signature, was observed across various datasets [8,12]. *RRP1B* has been represented as a likely biomarker for early gastric cancer. The expression level of *RRP1B* was significantly reduced in 76 early gastric cancer tissues compared with normal cases in the Chinese study [35]. The other study involved the analysis of 54 pairs of laryngeal tumor and adjacent normal tissues, it was revealed that *RRP1B* is significantly downexpressed in laryngeal squamous cell carcinoma [36]. There is a potential link between ALY (Aly/REF export factor), RRP1B, and metastasis in oral squamous cell carcinoma (OSCC). A knockdown of ALY reduces invasiveness and migration in OSCC cells, accompanied by an increase in *RRP1B* expression. Elevated RRP1B, alongside CD82, in ALY knockdown cells indicates that *RRP1B* may play a key role in regulating OSCC cellular invasiveness and migration [37].

Several studies have been conducted to evaluate the influence of *RRP1B* in non-oncological diseases. *RRP1B* is one of the genes regulating AREG (Amphiregulin) in endothelial cells, with HIF-1α playing a role in their upregulation in hypoxia. Silencing *RRP1B* reduces inflammation and apoptosis, highlighting its potential significance in pulmonary hypertension pathology [38]. It has been identified that *RRP1B* participates in the pathogenetic process of sepsis by regulating the activation and differentiation of lymphocytes. [39]. Based on a large-scale genome-wide association study, *RRP1B* is associated with a significant signal of blood pressure regulation [40]. The *RRP1B* gene was associated with blood pressure response to specific antihypertensive drugs, particularly atenolol [41]. The expression of *RRP1B* was analyzed in leucocytes of individuals with Down’s syndrome (DS). The results indicated that *RRP1B* showed significant upregulation in DS patients compared to the normal population [42].

It is interesting that data from the Cancer Genome Atlas (TCGA) project suggest the expression level of *RRP1B* is not a prognostic factor in cervical cancer survival analysis (https://tcga-data.nci.nih.gov/docs/publications/cesc_2016/, accessed on 15 December 2023).

There are few studies evaluating the associations of *RRP1B* rs9306160 polymorphisms with cancer risk or clinical data.

In our study, we found that the C allele of rs9306160 is more common and may have a significant protective effect against metastasis (*p* = 0.008). The variant T allele did not show statistically significant results, but the TT genotype increased the risk for metastasis (*p*-value close to the significance at 0.051). Unfortunately, we did not obtain significant associations between rs9306160 and clinical features such as lymph node metastasis, tumor differentiation, or survival rates. But if we consider that a frequent allele is a sign of a better prognosis, then when analyzing the results of other authors’ studies, the data differ.

Crawford et al.‘s study with breast cancer outcomes was conducted in two cohorts: one from Orange County and another from the Greater Baltimore Area. Consistent findings were observed between the cohorts, although some differences could be attributed to cohort characteristics. They found a significant association between the variant A allele of rs9306160 and disease stage in a Caucasian cohort. The A allele was more prevalent in patients with localized disease compared to those with advanced regional or metastatic disease. The variant allele showed significant associations with various tumor characteristics, including estrogen receptor (ER) and progesterone receptor (PR) status, the presence of lymph node disease, and tumor grade. It was more frequent in patients with ER-positive and PR-positive tumors, as well as in those with well-to-moderately differentiated tumors. Carriers of the variant allele had better breast cancer-specific survival compared to homozygous carriers of the common allele (G/G). This survival advantage was more pronounced in patients with ER-positive tumors [8].

Another study involved 1863 Dutch patients with operable primary breast cancer from Rotterdam, The Netherlands. The investigation identified a significant association of variants in rs9306160 with metastasis-free survival (MFS) (*p* = 0.012). Specifically, the study revealed a connection between the T allele of the *RRP1B* SNP (rs9306160) and a more favorable prognosis in MFS among breast cancer patients. Carrying the T allele (CT or TT genotypes) of rs9306160 was associated with a positive outcome in terms of MFS. Remarkably, this association maintained significance even in multivariate analysis, indicating that the T allele functions as an independent prognostic factor. Notably, the association with patients’ survival was confined to estrogen receptor-positive, lymph node-negative (ER+/LN−) patients (*p* = 0.011). Furthermore, combining the genotypes of two genes (*SIPA1* and *RRP1B*) demonstrated a significant ability to discriminate patients with poor metastasis-free survival (HR: 0.40, 95% CI: 0.24 to 0.68, *p* = 0.001). It is important to acknowledge the study’s limitations, as the observed association was significant only for a specific subgroup (ER+/LN− patients) and not for other patient subgroups (ER+/LN+, ER−/LN+, ER−/LN−). The study was conducted within a Dutch patient population, and to establish broader applicability, the results may require validation in diverse populations [43].

On the other hand, the study of Nanchari et al., which included 493 breast cancer cases and 558 age-matched healthy female controls, could reflect a guideline for the results we obtained. The TT genotype and T allele frequencies of the *RRP1B* rs9306160 (1307T>C) polymorphism were significantly elevated in breast cancer cases compared to controls. The presence of the T allele conferred a 1.75-fold increased risk for breast cancer development. The TT genotype was associated with a higher risk under codominant and recessive models. Moreover, the TT genotype frequency was significantly elevated in obese patients, patients with advanced disease, and those with increased tumor size. The T allele was associated with positive lymph node status and Her2-negative receptor status. In silico analysis of RNA secondary structures near the SNP site indicated that the T allele may result in a less stable mRNA structure compared to the C allele, potentially affecting functional interactions. The study suggests that the TT genotype may increase the risk for both breast cancer development and progression. It acknowledges deviations from the Hardy–Weinberg equilibrium and suggests the possibility of selective forces influencing genotype frequencies over generations. Additionally, the study highlights discrepancies in results compared to other cohorts, possibly due to ethnic variations. The C allele was more frequent in both controls and breast cancer cases, indicating that the C allele was more prevalent in both groups. However, there were differences in allele frequencies between controls and breast cancer cases. It is important to note that the findings are specific to the population studied (Southern Indian) and may not be directly applicable to other populations [44].

Earlier research from Lithuania characterized a group of young Lithuanian patients with breast cancer. Consistent with our findings, the prevalence of the C allele of rs9306160 (c.436T4C) was higher, constituting 59.5% in the allelic model. The study revealed a statistically significant association between rs9306160 and tumor grade (G). Specifically, the T allele was significantly linked to G3 tumor grade (high-grade tumors), indicating a higher probability of G3 grade in carriers of the T allele. This association remained significant after adjustments, including age at diagnosis, tumor receptor status, tumor size, and lymph node involvement, suggesting an independent effect of the polymorphism on this breast cancer characteristic. The C allele was associated with ER-positive status, implying a higher likelihood of positive ER in individuals with the CC genotype or carriers of the C allele. Therefore, these findings support the notion of the T allele as a worse prognostic factor [45].

Moreover, a case-control study involving 100 Iraqi women (75 with confirmed breast cancer and 25 with normal breast tissue) could also corroborate the observed trend in our results. The results indicated a higher frequency of the CC genotype in the control group. The homozygous TT genotype was associated with histologic grade, and this association remained significant across all grades. Among cancer patients with a high-grade variant, T alleles were more prevalent compared to those in low-grade conditions. Furthermore, the (TT) genotype was more frequently observed in breast cancer cases with metastatic lymph node involvement compared to cases without lymph node involvement [46].

Our extended haplotype analysis of the investigated SNPs revealed that GCTTC haplotype non-carriers, predominantly consisting of ATCCG haplotypes, were less likely to exhibit advanced tumor size and metastasis. These findings were consistent with the results obtained from allelic models. The same trend was also noted in survival assessments. Consequently, we posit that these haplotypes could serve as independent markers.

In the present study, we examined the associations between five functional SNPs in the *RRP1B* gene and the clinicopathological profiles and survival rates in a cohort of Lithuanian women with cervical cancer. Our study is the first to analyze *RRP1B* SNPs for assessing the clinicopathological features and progression of CC. It establishes a link between SNPs in *RRP1B* and CC, suggesting these genetic variants as predictive biomarkers for prognosticating the development of the disease in the future. The study boasts several strengths, including a comprehensive dataset comprising genetic data, tumor phenotype information, and survival data. However, certain limitations warrant consideration. Notably, the absence of comparable studies on associations between these polymorphisms and clinicopathological characteristics of CC prevents a direct comparison of our results. Additionally, the limited sample size may have influenced the robustness of our findings. Furthermore, a notable weakness is the absence of a control group, hindering the assessment of CC risk.

Our investigation indicates a potential link between *RRP1B* polymorphisms and the pathomorphological features of cervical cancer, as well as disease outcomes. The association of these genetic variations with the aggressiveness of cervical cancer underlines the importance of considering germline factors in understanding cancer behavior. This observation opens avenues for further research to elucidate the mechanistic basis of *RRP1B*’s involvement in metastatic processes and its clinical implications. While *RRP1B* may not traditionally be classified as an oncogene, we believe that its inclusion in our investigation offers a unique opportunity to uncover novel facets of the disease’s molecular underpinnings. Importantly, our decision to study *RRP1B* stems from a comprehensive approach aimed at elucidating the full spectrum of genetic factors contributing to cervical cancer development and progression. We recognize that the complexity of cancer biology extends beyond well-established oncogenes, and exploring genes like *RRP1B* allows us to broaden our understanding of the disease.

## 5. Conclusions

All investigated *RRP1B* polymorphisms (rs2838342, rs7276633, rs2051407, rs9306160, and rs762400) in our study have the potential to serve as markers for clinical characteristics and prognosis in cervical cancer. Among these, three (rs2051407, rs9306160, and rs762400) were found to be significant in relation to metastasis, while rs2838342 showed potential association with metastasis. Rs2838342, rs7276633, rs2051407, and rs762400 showed the assotiations with survival outcomes. Haplotypes analysis was in line with the allelic models. These results highlight the intricate interplay between genetic factors and clinical dynamics in the progression of tumors. Nevertheless, it is crucial to acknowledge that certain comparisons did not attain statistical significance, possibly owing to the relatively small sample size. Considering the clinical context is imperative, it is essential to interpret the results cautiously, especially for genotypes or alleles with borderline significance levels. Our results offer insights for subsequent studies on cervical cancer and other cancer types, examining these polymorphisms to ascertain their functionality. In the future, SNP detection in *RRP1B* may serve as a predictive tool for assessing the clinical manifestations and prognoses of cervical cancer. While evidence is accumulating regarding the significance of genetic variation in the etiology and development of cervical cancer, research exploring the role of metastasis-related gene variants in cervical cancer is still in its early stages. Further investigations are required to validate these observations and gain a comprehensive understanding of the underlying biological mechanisms.

## Figures and Tables

**Table 1:** SNP genomic region, minor allele frequency (MAF).

Chromosome/Gene	SNP	Genomic Position in Chromosome	Region/Location	Minor Allele Frequency (MAF) (1000 Genomes)	Highest Population MAF
Chr21/ RRP1B Alias symbols: KIAA0179, Nnp1 RRP1 PPP1R136	rs2838342	43657984 (GRCh38) 45077865 (GRCh37)	Upstream transcript variant, intron variant	0.42/(G)	0.50
	rs7276633	43658919 (GRCh38) 45078800 (GRCh37)	Upstream variant	0.42/(C)	0.49
	rs2051407	43659364 (GRCh38) 45079245 (GRCh37)	Upstream variant	0.37/(T)	0.42
	rs9306160	43687681 (GRCh38) 45107562 (GRCh37)	Missense variant	0.38/(T)	0.44
	rs762400	43693748 (GRCh38) 45113629 (GRCh37)	3′-UTR variant	0.37/(C)	0.50

GRCh37 and GRCh38 are human genome assembly versions by the Genome Reference Consortium.

**Table 2:** The distribution of *RRP1B* genotypes and alleles.

SNP	Sample Size N (Study Cohort)	Genotypes	Genotypes Frequencies		Alleles	Alleles Frequencies (Study Cohort)	Sample Size N (1000 Genomes)	Alleles Frequencies (1000 Genomes)	MAF *p* Value ^a^
rs2838342 A>G	172	AA AG GG	58/33.7% 85/49.4% 29/16.9%	0.337 0.494 0.169	A G	0.584 0.416	1006	0.589 0.420	0.791
rs7276633 T>C	172	TT TC CC	59/34.3% 84/48.8% 29/16.9%	0.343 0.488 0.169	T C	0.587 0.413	1006	0.581 0.420	0.676
rs2051407 C>T	172	CC CT TT	63/36.6% 84/48.8% 25/14.5%	0.366 0.489 0.145	C T	0.610 0.390	1006	0.634 0.366	0.282
rs9306160 * C>T	169	CC CT TT	55/32.5% 92/54.4% 22/13.0%	0.325 0.545 0.130	C T	0.598 0.402	1006	0.617 0.383	0.450
rs762400 G>C	172	GG GC CC	63/26.6% 88/51.2% 21/12.2%	0.366 0.512 0.122	G C	0.622 0.378	1006	0.626 0.374	0.804

* Genotypes were determined among 169 patients due to non-amplification in three cases. ^a^ A chi-squared test for independence analysis for the number of each minor allele in cases and controls.

**Table 3:** Haplotypes and frequencies.

Haplotype Number	rs2838342 rs7276633 rs2051407 rs9306160 rs762400 Haplotypes	Chromosomes (Counts)	Frequencies (%)
1	ATCCG	190	55.23
2	ATCTG	5	1.45
3	ATCTC	2	0.58
4	ATTCG	1	0.29
5	ACCCG	2	0.58
6	GTCCG	1	0.29
7	GTTTC	1	0.29
8	GCCCG	4	1.16
9	GCCTG	5	1.45
10	GCCTC	1	0.29
11	GCTCG	6	1.74
12	GCTCC	2	0.58
13	GCTTC	124	36.05

Thirteen haplotypes were generated from the analyzed SNPs through the Phase software v2.1.

**Table 4:** Univariate and multivariate logistic regression analyses were conducted for rs2838342, adjusting for alleles, and clinicopathological characteristics, with a focus on tumor size and metastasis.

			Univariate			Multivariate											
SNP	Dependent	Covariates				Model No. 1			Model No. 2			Model No. 3			Model No. 4		
			Odds	95% CI	*p*	Odds	95% CI	*p*	Odds	95% CI	*p*	Odds	95% CI	*p*	Odds	95% CI	*p*
rs2838342	Positive T3-T4	A allele + vs. A −	0.281	0.122–0.643	0.002	0.280	0.122–0.643	0.003	0.299	0.129–0.692	0.005	0.244	0.096–0.619	0.003	0.266	0.102–0.691	0.007
		Age (years)				1.001	0.978–1.025	0.909	1.002	0.979–1.027	0.842	1.028	1.000–1.057	0.054	1.027	0.999–1.056	0.059
		Positive G3 vs. G1 + G2							1.991	0.978–4.051	0.058	1.798	0.826–3.914	0.140	1.687	0.762–3.732	0.197
		Positive N1 vs. N0										7.367	3.347–16.217	<0.001	6.161	2.756–13.771	<0.001
		Positive M1 vs. M0													5.977	0.690–51.748	0.105
rs2838342	Positive M	A allele + vs. A −	0.274	0.072–1.040	0.044	0.272	0.071–1.140	0.057	0.291	0.075–1.127	0.074	0.267	0.063–1.136	0.074	0.521	0.124–2.190	0.374
		Age (years)				0.979	0.932–1.028	0.395	0.980	0.933–1.029	0.417	1.009	0.960–1.061	0.714	0.978	0.927–1.031	0.401
		Positive G3 vs. G1 + G2							1.603	0.416–6.170	0.493	1.293	0.315–5.308	0.721	1.130	0.276–4.633	0.865
		Positive N1 vs. N0										0.000	0.000	0.996	0.000	0.000	0.996
		Positive T3–T4													15.623	1.853–131.722	0.012

**Table 5:** Univariate logistic regression analysis: assessing odds ratios for the relationships between SNPs and patients’ age, cancer stage groups, and disease prognosis.

		Age (Groups): ≤50 vs. >50			Positive Stage III–IV vs. Stage I–II			Positive Worse Prognosis: T3–T4 + G3 vs. T1–T2 + G1–G2		
SNP	Genotype, Alleles	OR	95% CI	*p*-Value	OR	95% CI	*p*-Value	OR	95% CI	*p*-Value
rs2838342	AG vs. AA	0.471	0.226–0.983	0.045						
	GG vs. AA									
	A allele + vs. A −				0.341	0.137–0.849	0.017	0.182	0.061–0.538	0.002
	G allele + vs. G −									
rs7276633	TC vs. TT									
	CC vs. TT									
	T allele + vs. T −				0.341	0.137–0.849	0.021	0.182	0.061–0.538	0.002
	C allele + vs. C −	2.138	1.080–4.230	0.029						
rs2051407	CT vs. CC									
	TT vs. CC									
	C allele + vs. C −							0.267	0.087–0.823	0.021
	T allele + vs. T −									

**Table 6:** Univariate and multivariate logistic regression analyses were conducted for rs7276633, adjusting for alleles and clinicopathological characteristics, with a focus on tumor size.

			Univariate			Multivariate											
SNP	Dependent	Covariates				Model No. 1			Model No. 2			Model No. 3			Model No. 4		
			Odds	95% CI	*p*	Odds	95% CI	*p*	Odds	95% CI	*p*	Odds	95% CI	*p*	Odds	95% CI	*p*
rs7276633	Positive T3–T4	T allele + vs. T −	0.281	0.122–0.643	0.003	0.280	0.122–0.643	0.003	0.299	0.129–0.692	0.005	0.277	0.125–0.708	0.003	0.264	0.147–0.893	0.007
		Age (years)				1.001	0.978–1.025	0.909	1.002	0.979–1.027	0.842	1.028	1.000–1.057	0.054	1.027	0.999–1.056	0.059
		Positive G3 vs. G1 + G2							1.991	0.978–4.051	0.058	1.798	0.826–3.914	0.140	1.687	0.762–3.732	0.197
		Positive N1 vs. N0										7.367	3.347–16.217	<0.001	6.161	2.756–13.771	<0.001
		Positive M1 vs. M0													5.977	0.690–51.748	0.105

**Table 7:** Univariate and multivariate logistic regression analyses were conducted for rs2051407, adjusting for alleles and clinicopathological characteristics, with a focus on tumor size and metastasis.

			Univariate			Multivariate											
SNP	Dependent	Covariates				Model No. 1			Model No. 2			Model No. 3			Model No. 4		
			Odds	95% CI	*p*	Odds	95% CI	*p*	Odds	95% CI	*p*	Odds	95% CI	*p*	Odds	95% CI	*p*
rs2051407	Positive T3–T4	C allele + vs. C −	0.393	0.166–0.929	0.033	0.392	0.166–0.928	0.033	0.414	0.173–0.992	0.048	0.354	0.134–0.930	0.035	0.409	0.149–1.123	0.083
		Age (years)				1.002	0.978–1.025	0.890	1.003	0.979–1.027	0.820	1.028	1.000–1.057	0.052	1.027	0.000–1.055	0.060
		Positive G3 vs. G1 + G2							2.067	1.027–4.161	0.042	1.885	0.877–4.050	0.104	1.803	0.827–3.928	0.138
		Positive N1 vs. N0										6.993	3.233–15.125	<0.001	5.795	2.639–12.726	<0.001
		Positive M1 vs. M0													6.116	0.713–52.493	0.099
rs2051407	Positive M	C allele + vs. C −	0.223	0.058–0.858	0.019	0.223	0.058–0.863	0.030	0.236	0.060–0.920	0.038	0.209	0.048–0.913	0.037	0.355	0.083–1.510	0.355
		Age (years)				0.979	0.931–1.029	0.403	0.980	0.932–1.031	0.432	1.007	0.957–1.059	0.801	0.979	0.926–1.034	0.443
		Positive G3 vs. G1 + G2							1.627	0.421–6.285	0.480	1.307	0.315–5.429	0.712	1.088	0.262–4.514	0.908
		Positive N1 vs. N0										0.000	0.000	0.996	0.000	0.000	0.996
		Positive T3–T4													15.475	1.852–129.314	0.011

**Table 8:** Univariate and multivariate logistic regression analyses were conducted for rs9306160, adjusting for alleles and clinicopathological characteristics, with a focus on metastasis.

			Univariate			Multivariate											
SNP	Dependent	Covariates				Model No. 1			Model No. 2			Model No. 3			Model No. 4		
			Odds	95% CI	*p*	Odds	95% CI	*p*	Odds	95% CI	*p*	Odds	95% CI	*p*	Odds	95% CI	*p*
rs9306160	Positive M	C allele + vs. C −	0.179	0.044–0.721	0.008	0.187	0.046–0.760	0.019	0.166	0.039–0.702	0.015	0.151	0.032–0.717	0.017	0.262	0.059–1.170	0.079
		Age (years)				0.979	0.929–1.032	0.430	0.981	0.931–1.034	0.479	1.002	0.949–1.059	0.932	0.977	0.921–1.036	0.437
		Positive G3 vs. G1 + G2							2.623	0.629–10.932	0.186	2.193	0.482–9.992	0.310	1.581	0.363–6.897	0.542
		Positive N1 vs. N0										0.000	0.000	0.996	0.000	0.000	0.996
		Positive T3–T4													12.411	1.442–106.842	0.002

**Table 9:** Univariate and multivariate logistic regression analyses were conducted for rs762400, adjusting for alleles and clinicopathological characteristics, with a focus on tumor size and metastasis.

			Univariate			Multivariate											
SNP	Dependent	Covariates				Model No. 1			Model No. 2			Model No. 3			Model No. 4		
			Odds	95% CI	*p*	Odds	95% CI	*p*	Odds	95% CI	*p*	Odds	95% CI	*p*	Odds	95% CI	*p*
rs762400	Positive T3–T4	G allele + vs. G −	0.383	0.151–0.967	0.037	0.383	0.151–0.968	0.042	0.378	0.148–0.970	0.043	0.330	0.115–0.946	0.039	0.401	0.132–1.216	0.106
		Age (years)				1.001	0.977–1.024	0.963	1.002	0.978–1.026	0.884	1.026	0.998–1.055	0.064	1.025	0.998–1.054	0.072
		Positive G3 vs. G1 + G2							2.173	1.080–4.372	0.029	2.008	0.933–4.320	0.074	1.906	0.877–4.143	0.103
		Positive N1 vs. N0										6.875	3.190–14.820	<0.001	5.717	2.612–12.511	<0.001
		Positive M1 vs. M0													5.895	0.682–50.958	0.107
rs762400	Positive M	G allele + vs. G −	0.176	0.045–0.686	0.006	0.165	0.042–0.659	0.011	0.168	0.042–0.673	0.012	0.149	0.032–0.703	0.016	0.265	0.062–1.135	0.074
		Age (years)				0.974	0.924–1.027	0.327	0.976	0.926–1.029	0.370	0.999	0.946–1.054	0.957	0.977	0.924–1.034	
		Positive G3 vs. G1 + G2							1.796	0.460–7.017	0.400	1.332	0.314–5.647	0.697	1.196	0.289–4.951	0.805
		Positive N1 vs. N0										0.000	0.000	0.996	0.000	0.000	0.996
		Positive T3–T4													14.735	1.757–123.541	0.013

**Table 10:** Univariate logistic regression analysis: assessing odds ratios for the relationships between diplotypes and patients’ age and tumor characteristics.

	Diplotypes														
Cinical Characteristics	Heterozygous Diplotype (ATCCG/Alternative Hap) vs. Homozygous Diplotype (ATCCG/ATCCG)			ATCCG Haplotype Non-Carriers vs. Homozygous Diplotype (ATCCG/ATCCG)			Heterozygous Diplotype (GCTTC/Alternative Hap) vs. Homozygous Diplotype (GCTTC/GCTTC)			GCTTC Haplotype Non-Carriers vs. Homozygous Diplotype (GCTTC/GCTTC)			Heterozygous Diplotype (ATCCG/GCTTC) Carriers vs. Non-Carriers		
	OR	95% CI	*p*-Value	OR	95% CI	*p*-Value	OR	95% CI	*p*-Value	OR	95% CI	*p*-Value	OR	95% CI	*p*-Value
Positive T3–T4 vs. T1–T2	0.383	0.183–0.800	0.077	1.773	0.703–4.471	0.225	0.506	0.185–1.387	0.186	0.367	0.136–0.992	0.038	0.424	0.220–0.817	0.090
Positive N1 vs. N0	*	*	*	*	*	*	0.831	0.311–2.221	0.712	1.222	0.449–3.326	0.694	0.671	0.364–1.238	0.202
Positive M1 vs. M0	1.022	0.180–5.790	0.980	3.538	0.606–20.653	0.160	0.250	0.056–1.110	0.068	0.098	0.016–0.578	0.010	0.313	0.064–1.517	0.149
Positive G3 vs. G1 + G2	0.699	0.317–1.538	0.373	1.406	0.534–3.705	0.491	1.271	0.417–3.876	0.673	0.923	0.290–2.935	0.892	0.908	0.458–1.801	0.783
Age (groups): ≤50 vs. >50	2.278	1.070–4.846	0.073	1.529	0.578–4.040	0.392	0.750	0.225–2.495	0.639	0.458	0.138–1.527	0.204	1.840	0.919–3.684	0.085
Positive stage III–IV vs. stage I–II	1.002	0.169–1.733	0.091	1.250	0.454–3.444	0.032	0.429	0.150–1.222	0.113	0.612	0.210–1.787	0.369	0.494	0.267–0.912	0.084
Positive worse prognosis: T3–T4 + G3 vs. T1–T2 + G1–G2	0.212	0.064–0.707	0.162	2.100	0.638–6.916	0.048	0.465	0.117–1.855	0.278	0.354	0.126–2.098	0.354	0.305	0.104–0.895	0.101

* OR could not be estimated because of zero value within a cell.

**Table 11:** Multivariate logistic regression analyses were conducted for diplotypes, adjusting for clinicopathological characteristics, with a focus on tumor size and metastasis.

			Multivariate											
SNPs	Dependent	Covariates	Model No. 1			Model No. 2			Model No. 3			Model No. 4		
			Odds	95% CI	*p*	Odds	95% CI	*p*	Odds	95% CI	*p*	Odds	95% CI	*p*
rs2838342 rs7276633 rs2051407 rs9306160 rs762400	Positive T3–T4													
		GCTTC haplotype non-carriers vs. homozygous diplotype (GCTTC/GCTTC)	0.393	0.188–0.822	0.039	0.392	0.185–0.827	0.041	0.391	0.173–0.884	0.041	0.380	0.166–0.869	0.046
		Age (years)	1.004	0.981–1.032	0.614	1.006	0.983–1.034	0.567	1.031	1.002–1.063	0.131	1.032	1.002–1.064	0.132
		Positive G3 vs. G1 + G2				2.007	0.971–4.147	0.061	1.842	0.831–4.067	0.131	0.579	0.257–1.299	0.185
		Positive N1 vs. N0							0.000	0.000	0.996	0.000	0.000	0.996
		Positive M1 vs. M0										6.508	0.719–58.708	0.096
rs2838342 rs7276633 rs2051407 rs9306160 rs762400	Positive M	GCTTC haplotype non-carriers vs. homozygous diplotype (GCTTC/GCTTC)	0.101	0.017–0.598	0.012	0.095	0.016–0.577	0.011	0.075	0.011–0.534	0.010	0.150	0.023–0.965	0.048
		Age (years)	0.980	0.927–1.035	0.462	0.983	0.931–1.039	0.544	1.005	0.952–1.062	0.850	0.982	0.926–1.041	0.545
		Positive G3 vs. G1 + G2				2.051	0.515–8.170	0.309	1.657	0.377–7.294	0.504	1.324	0.312–5.579	0.702
		Positive N1 vs. N0							0.000	0.000	0.996	0.000	0.000	0.996
		Positive T3–T4										8.404	0.915–77.157	0.060

**Table 12:** Cox’s univariate model for PFS and OS.

			Progression-Free Survival			Overall Survival	
SNP	Genotype/Allele	HR	95% CI	*p*-Value	HR	95% CI	*p*-Value
rs2838342	AG vs. AA	0.560	0.303–1.036	0.065	0.742	0.358–1.539	0.423
	GG vs. AA	1.155	0.545–2.447	0.706	1.824	0.827–4.025	0.137
	A allele + vs. A −	1.572	0.784–3.151	0.202	0.465	0.232–0.931	0.031
	G allele + vs. G −	0.682	0.391–1.187	0.176	0.990	0.516–1.899	0.977
rs7276633	TC vs. TT	0.577	0.312–1.066	0.079	0.763	0.368–1.582	0.467
	CC vs. TT	1.176	0.555–2.490	0.673	1.854	0.840–4.090	0.126
	T allele + vs. T −	0.636	0.317–1.275	0.202	0.465	0.232–0.931	0.031
	C allele + vs. C −	0.700	0.402–1.219	0.208	1.015	0.529–1.946	0.964
rs2051407	CT vs. CC	0.556	0.302–1.026	0.060	0.813	0.397–1.667	0.573
	TT vs. CC	1.314	0.604–2.842	0.488	2.144	0.959–4.793	0.063
	C allele + vs. C −	0.568	0.275–1.175	0.127	0.418	0.204–0.858	0.017
	T allele + vs. T −	0.689	0.397–1.194	0.184	1.081	0.568–2.056	0.812
rs9306160	CT vs. CC	0.647	0.352–1.187	0.160	0.856	0.418–1.751	0.669
	TT vs. CC	1.357	0.595–3.092	0.468	2.213	0.943–5.193	0.068
	C allele + vs. C −	0.613	0.286–1.314	0.208	0.498	0.229–1.084	0.079
	T allele + vs. T −	0.705	0.399–1.245	0.228	0.915	0.476–1.758	0.789
rs762400	GC vs. GG	0.613	0.336–1.117	0.110	0.917	0.451–1.866	0.811
	CC vs. GG	1.443	0.642–3.240	0.375	2.550	1.098–5.923	0.030
	G allele + vs. G −	0.537	0.251–1.147	0.108	0.374	0.177–0.788	0.010
	C allele + vs. C −	0.734	0.422–1.275	0.272	1.178	0.613–2.263	0.624

**Table 13:** Cox’s multivariate models for overall survival: adjusted ratios for associations between SNPs, age at diagnosis, and tumor characteristics.

	Variables	Overall Survival		
		HR	95% CI	*p*-Value
rs2838342	A allele + vs. A −	0.462	0.231–0.926	0.030
Model No. 1	Age at diagnosis	1.011	0.987–1.037	0.366
	A allele + vs. A −	0.802	0.382–1.686	0.561
rs2838342	Age at diagnosis	1.020	0.995–1.046	0.110
Model No. 2	T3–T4 vs. T1–T2	7.463	3.195–17.432	<0.001
	N1 vs. N0	1.874	0.907–3.872	0.090
	G3 vs. G1–G2	0.710	0.346–1.457	0.350
rs7276633	T allele + vs. T −	0.462	0.231–0.926	0.030
Model No. 1	Age at diagnosis	1.011	0.987–1.037	0.366
rs7276633	T allele + vs. T −	0.802	0.382–1.686	0.561
Model No. 2	Age at diagnosis	1.029	0.995–1.046	0.110
	T3–T4 vs. T1–T2	7.463	3.195–17.432	<0.001
	N1 vs. N0	1.874	0.907–3.872	0.090
	G3 vs. G1–G2	0.710	0.346–1.457	0.350
rs2051407	C allele + vs. C −	0.404	0.196–0.832	0.014
Model No. 1	Age at diagnosis	1.013	0.988–1.039	0.297
Rs2051407	C allele + vs. C −	0.604	0.285–1.281	0.189
Model No. 2	Age at diagnosis	1.022	0.997–1.048	0.082
	T3–T4 vs. T1–T2	7.484	3.227–17.355	<0.001
	N1 vs. N0	1.824	0.892–3.732	0.100
	G3 vs. G1–G2	0.698	0.346–1.405	0.313
rs762400	GC vs. GG	0.858	0.416–1.767	0.677
Model No. 1	CC vs. GG	2.476	1.064–5.758	0.035
	Age at diagnosis	1.013	0.987–1.040	0.325
rs762400	GC vs. GG	1.083	0.521–2.248	0.831
Model No. 2	CC vs. GG	1.865	0.785–4.431	0.158
	Age at diagnosis	1.021	0.996–1.047	0.100
	T3–T4 vs. T1–T2	7.546	3.250–17.520	<0.001
	N1 vs. N0	1.814	0.882–3.731	0.105
	G3 vs. G1–G2	0.719	0.358–1.448	0.356
rs762400	G allele + vs. G −	0.370	0.176–0.781	0.009
Model No. 1	Age at diagnosis	1.012	0.987–1.038	0.356
rs762400	G allele + vs. G −	0.560	0.261–1.203	0.137
Model No. 2	Age at diagnosis	1.021	0.996–1.047	0.100
	T3–T4 vs. T1–T2	7.496	3.235–17.373	<0.001
	N1 vs. N0	1.798	0.879–3.677	0.108
	G3 vs. G1–G2	0.728	0.365–1.452	0.367

**Table 14:** Cox’s univariate model for PFS and OS.

		Progression-Free Survival			Overall Survival	
*RRP1B* Haplotypes	HR	95% CI	*p*-Value	HR	95% CI	*p*-Value
Heterozygous diplotype (ATCCG/alternative haplotype) vs. homozygous diplotype (ATCCG/ATCCG)	0.990	0.523–1.873	0.975	0.843	0.397–1.790	0.656
ATCCG haplotype non-carriers vs. homozygous diplotype (ATCCG/ATCCG)	2.244	0.991–5.080	0.052	2.121	0.910–4.943	0.081
Heterozygous diplotype (GCTTC/alternative haplotype) vs. homozygous diplotype (GCTTC/GCTTC)	0.485	0.208–1.132	0.094	0.274	0.120–0.626	0.002
GCTTC haplotype non-carriers vs. homozygous diplotype (GCTTC/GCTTC)	0.434	0.190–0.993	0.051	0.298	0.128–0.695	0.005
Heterozygous diplotype (ATCCG/GCTTC) carriers vs. non-carriers	0.872	0.479–1.588	0.655	0.694	0.362–1.331	0.271

**Table 15:** Cox’s multivariate models for overall survival: adjusted ratios for associations between diplotypes, age at diagnosis, and tumor characteristics.

SNPs	Covariates	Model No. 1			Model No. 2		
		Odds	95% CI	*p*	Odds	95% CI	*p*
rs2838342 rs7276633 rs2051407 rs9306160 rs762400	Heterozygous diplotype (GCTTC/alternative hap) vs. homozygous diplotype (GCTTC/GCTTC)	0.259	0.113–0.597	0.002	0.372	0.153–0.904	0.029
	GCTTC haplotype non-carriers vs. homozygous diplotype (GCTTC/GCTTC)	0.303	0.130–0.708	0.006	0.363	0.151–0.871	0.023
	Age (years)	1.013	0.987–1.039	0.334	1.027	1.000–1.054	0.050
	G3 vs. G1–G2				0.760	0.374–1.547	0.449
	N1 vs. N0				1.913	0.933–3.922	0.076
	T3–T4 vs. T1–T2				7.412	3.196–17.188	<0.001

## Data Availability

The data presented in this study are available on request from the corresponding author.
